# 

**Published:** 1880-02

**Authors:** 


					﻿Whole Number
ccecxvn.
February, 1880.
Number 2,
Vol. XL.
THE
OTTICJVGO
MEDIC AL
T ournal and Examiner
EDITORS:
N. S. DAVIS, M.D., LL.D. JAS. NEVINS HYDE, A.M., M.D.
DANIEL R. BROWER, M.D.
CHICAGO:
PUBLISHED BY THE CHICAGO MEDICAL PRESS ASSOCIATION.
188 Clark Street.
i^'Read the Notice of this Journal amone Advertisements.
				

